# Reevaluating methods reporting practices to improve reproducibility: an analysis of methodological rigor for the Langendorff whole heart technique

**DOI:** 10.1152/ajpheart.00164.2022

**Published:** 2022-06-24

**Authors:** D. Ryan King, Kathryn M. Hardin, Gregory S. Hoeker, Steven Poelzing

**Affiliations:** ^1^Translational Biology, Medicine, and Health Graduate Program, Virginia Polytechnic Institute and State University, Blacksburg, Virginia; ^2^Dorothy M. Davis Heart and Lung Research Institute, College of Medicine, The Ohio State University Wexner Medical Center, Columbus, Ohio; ^3^Virginia Tech Carilion School of Medicine, Roanoke, Virginia; ^4^Center for Heart and Reparative Medicine Research, Fralin Biomedical Research Institute at Virginia Tech Carilion, Roanoke, Virginia; ^5^Department of Biomedical Engineering and Mechanics, Virginia Polytechnic Institute and State University, Blacksburg, Virginia

**Keywords:** electrophysiology, Langendorff, methods reporting, reproducibility

## Abstract

In recent decades, the scientific community has seen an increased interest in rigor and reproducibility. In 2017, concerns about methodological thoroughness and reporting practices were implicated as significant barriers to reproducibility within the preclinical cardiovascular literature, particularly in studies using animal research. The Langendorff, whole heart technique has proven to be an invaluable research tool, being modified in a myriad of ways to probe questions across the spectrum of physiological and pathophysiological functions of the heart. As a result, significant variability in the application of the Langendorff technique exists. This literature review quantifies the different methods employed in the implementation of the Langendorff technique and provides brief examples of how individual parametric differences can impact the outcomes and interpretation of studies. From 2017 to 2020, significant variability of animal models, anesthesia, cannulation time, perfusate composition, pH, and temperature demonstrate that the technique has diversified to meet new challenges and answer different scientific questions. The review also reveals which individual methods are most frequently reported, even if there is no explicit agreement upon which parameters should be reported. The analysis of methods related to the Langendorff technique suggests a framework for considering methodological approach when interpreting seemingly contradictory results, rather than concluding that results are irreproducible.

## INTRODUCTION

In recent decades, the scientific community has seen an increased interest in rigor and reproducibility, as is evident by an increase in the number of studies investigating the perceived lack of scientific reproducibility (1). A precipitating event leading to the renewed interest in reproducibility can be traced back to the United States Food and Drug Administration’s withdrawal of 10 prescription drugs from the market between 1997 and 2000. These steps were taken after the drugs’ adverse effects in females became apparent, effects which were not detected in preclinical research nor clinical trials as a result of the male bias present in those studies (GAO-01–754) (2).In response to the historical male bias in scientific studies, the National Institutes of Health (NIH) Revitalization Act, PL 103-43 directed the NIH to establish guidelines for the inclusion of females in clinical research in 1993. In June 2015, the NIH further released notice NOT-OD-15–102, requiring consideration of sex as a biological variable in preclinical research funded by the agency.

Also contributing to the interest in scientific reproducibility, Bayer HealthCare and Amgen published reports in which they were only able to reproduce results from less than 30% of selected high-impact preclinical studies (3, 4). This added fuel to a “reproducibility crisis” narrative in both the scientific and public spheres. Since the release of these reports, there has been an increase in the number of popular editorials, commentaries, and books targeted to lay audiences to raise awareness (and sometimes stoke fear/outrage) among the general public regarding the impact of irreproducibility in federally funded biomedical research (5–7).

In 2017, our field was brought into the fold when “poor methodological rigor and [lack of] transparent reporting practices” were implicated as significant impediments to reproducibility within the preclinical cardiovascular literature (8). In this review, we summarize the state of methods reporting for the 5 years following the publication of NIH NOT-OD-15–102 as it relates specifically to the Langendorff technique, given its continued widespread use in preclinical cardiac research and our laboratory’s continued use of the technique for basic science investigations.

First performed in 1866 by Carl Ludwig and Elias Cyon, and modified into modern form in 1898 by Oskar Langendorff, the retrograde perfusion of the ex vivo heart has been instrumental in advancing our knowledge of cardiac physiology (9–13). In the past century, the Langendorff technique, including the perfusion systems and fluids paired with it, has been adapted for use in a wide variety of preclinical animal models (10, 14, 15). In this article, the evolution of experimental design(s) associated with the Langendorff technique and the potential impact of these variations to alter experimental outcomes are detailed and discussed. Although the review is highly focused on one particular technique that is also narrowly confined to only a few fields of scientific inquiry, the case study is illustrative of the lack of or evolution of unstated methods reporting consensus. The review also offers a handful of peer-reviewed example studies for why a particular variable may be worth reporting. Finally, a few thoughts are offered on how methods reporting could be improved by deliberate conversation.

## METHODS

A systematic review of the scientific literature was performed on July 29, 2021. The search was conducted in PubMed Central (http://www.ncbi.nlm.nih.gov/pmc) using the search terms: (Isolated[All Fields] AND (“heart”[MeSH Terms] OR “heart”[All Fields])) AND (Retrograde[All Fields] AND (“perfusion”[MeSH Terms] OR “perfusion”[All Fields]))) OR Langendorff[All Fields] AND (“open access”[filter] AND (“2016/01/01”[PubDate]: “2020/12/31”[PubDate]). In other words, this expression searches for manuscripts that contain the following keywords: Isolated Heart and Retrograde Perfusion, Isolated Heart and Perfusion, Isolated Heart and Langendorff, Heart and Retrograde Perfusion, Heart and Perfusion, and Heart and Langendorff. The query identified 2,597 manuscripts.

### Characteristics of Studies Included

This review only includes experimental studies that use the Langendorff technique for primary data collection. Exclusion criteria were as follows: manuscripts that were not primary research articles (i.e., editorials, reviews, case studies, etc.), manuscripts that only used Langendorff perfusion for the purpose of cell isolation, and manuscripts that used only working heart preparations. Following exclusions on the aforementioned principles, 443 manuscripts (*n*) were included in the analysis.

#### Data collection instrument.

The outcomes of the PubMed Central search were collected in a standardized data collection record that contained the following fields: PMCID, Journal Name, Animal Species, Animal Sex, Animal Age, Animal Weight, Organ Weight, Anesthesia Use (yes/no), Heparin Use (yes/no), Cannulation Time, Experimental Temperature, Perfusate Name, Perfusate pH, Perfusate Recirculation, Perfusate Composition Provided (yes/no), [NaCl], [NaHCO_3_], [NaH_2_PO_4_], [NaOH], [NaSO_4_], [Na_2_EDTA], [Na-Hexanoate], [KCl], [KH_2_PO_4_], [KHCO_3_], [MgSO_4_], [MgCl_2_], [d-glucose], [CaCl_2_], [H_2_PO_4_], [HEPES], [Pyruvate], [Na-Pyruvate], [EDTA], [Acetate], [Octanoic Acid], [Oleate], [Insulin], [Bovine Serum Albumin], [2,3-butanedione monoxime], [Blebbistatin], [Lactate], [Taurine].

The database of results has been uploaded to figshare.com and can be accessed with the unique Digital Object Identifier (DOI: https://doi.org/10.6084/m9.figshare.20137562).

## RESULTS

### Animal Model

An important consideration in preclinical research is the choice of experimental model. For research involving animal studies, factors to consider include utility as a research model, level of clinical translatability, cost, and maintenance. The choice of model extends beyond species to include the decision of genetic strain, sex, age, and weight.

#### Species.

Of the 443 manuscripts meeting inclusion criteria for this review, the majority of studies used rodent models for studying cardiac function with the Langendorff technique: 125 mouse, 252 rat, 38 rabbit, 15 guinea pig, 4 canine, 11 pig, 1 chicken, 1 hamster, 1 sheep, and 1 trout (Tables 1 and 2). Six manuscripts reported data from multiple animal species, which have all been independently quantified in the aforementioned list. Although larger mammalian species may serve as closer homologs to humans in certain regard, they often lack the utility found in smaller species such as rodents. This point is illustrated by the insight into genomic regulation of phenotype provided by the use of transgenic mouse models, despite the fact that murine models have substantial differences in cardiac ultrastructure, ion exchange, and protein expression relative to humans (16–19). For instance, calcium handling differs greatly across species; small rodents have an increased dependence on the sarco/endoplasmic reticulum Ca^2+^-ATPase for relaxation, as opposed to the cardiac sodium-calcium exchanger (NCX) (20–23). There are also appreciable differences in potassium channel expression and potassium current densities that result in heterogeneous repolarization patterns and action potential morphologies (24, 25). Specifically, in mouse and rat, the large transient outward potassium (K^+^) current (I_to_) is predominantly responsible for rapid membrane repolarization, whereas in human, rabbit, canine, and guinea pig, the delayed rectifier K^+^-currents, I_Ks_ and I_Kr_, are primarily responsible for membrane repolarization (24–26). Guinea pigs and pigs on the other hand do not functionally express I_to_, which has been implicated as a major cause of early repolarization in diseases such as the Brugada syndrome (27–29). Thus, species differences can be important when studying intracellular calcium handling or repolarization. Given the preponderance of rodent studies in the field, the remainder of the review will mainly provide exemplars of how rodent-specific physiology is affected by choice of experimental technique.

**Table 1.** Description of animal sex reporting broken down by animal species

Animal Sex	Chicken	Dog	Guinea Pig	Hamster	Mouse	Pig	Rabbit	Rat	Sheep	Trout	Totals	% of Total
Male	0	2	8	1	59	0	13	201	0	0	284	63.3
Female	0	0	4	0	4	0	4	11	0	0	23	5.1
Both	0	0	3	0	22	1	12	14	0	0	52	11.6
Not provided	1	2	0	0	40	10	9	26	1	1	90	20.0
Totals	1	4	15	1	125	11	38	252	1	1	449	100.0

**Table 2.** Description of animal age and weight reporting broken down by animal species

	Chicken	Dog	Guinea Pig	Hamster	Mouse	Pig	Rabbit	Rat	Sheep	Trout	Totals	%
Age	0	3	10	1	5	8	28	126	1	1	183	40.8
Weight	0	0	2	0	74	0	1	37	0	0	114	25.4
Both	0	1	1	0	12	0	4	70	0	0	88	19.6
Not provided	1	0	2	0	34	3	5	19	0	0	64	14.2
Totals	1	4	15	1	125	11	38	252	1	1	449	100.0

#### Genetic strain.

The genetic strain of animals was not always readily apparent in the manuscripts analyzed. In many cases, the strain was determined by following several citations to the original studies describing species strain background. In these cases, it was not always clear whether or not the transgenic mouse had been maintained on the original background or bred into a new strain.

Within a given species, differences in experimental outcomes have been linked to variation among genetic strains (30, 31). For instance, Maudsley nonreactive rats display a faster decline in norepinephrine levels in their heart than reactive strains, hinting at a strain-specific determinant to norepinephrine metabolism, which is important given the frequent use of isoproterenol, the isopropylamine analog of epinephrine, in Langendorff preparations (32). Jelinek et al. (31) further demonstrated that genetic background is the principal determinant of ventricular arrhythmia susceptibility following ß-adrenergic stimulation in mice. Furthermore, Obergassel et al. (33) used Langendorff-perfused mouse hearts to elegantly demonstrate that genetic strain has a strong role in atrial electrophysiological parameters including action potential duration, interatrial activation times, and atrial effective refractory periods.

These variances in outcomes extend to environmental factors surrounding how the species are housed and cared for leading up to experiments. For example, the development of myocardial lesions in response to a high-fat diet is mouse strain-specific, suggesting that studies within the same species may not be directly comparable without consideration of genetic strain (34). This becomes particularly important with varying ages and diets of the study animals.

#### Sex.

In the manuscripts reviewed, 63.3% (*n* = 284) included only male animals in their studies, 5.1% (*n* = 23) included only females, 11.6% (*n* = 52) included both males and females, whereas 20.0% (*n* = 90) did not report the sex of the animals used (Table 1).

Sex as a biological variable is well known to affect cardiac function in both physiological and pathophysiological states. Recently, the *American Journal of Physiology - Heart and Circulatory Physiology* editorial staff published an article outlining the importance of and reinforcing the journal’s commitment to, the inclusion of sex as a biological variable (35). Numerous recent reports provide further evidence for the importance of sex-specific signaling leading to differences in electrophysiological, mechanical, and metabolic function of the heart (36–41). For example, different levels of endogenous estrogen receptor signaling are known to lead to sex-based differences in genomic expression of the α subunit of the L-type Ca^2+^ channel and NCX1, expression and activity of the Ryanodine receptor (RyR2), ejection fraction and strength of contraction, and rate of repolarization (QT interval) (42–51). Although intrinsic neurohumoral modification of cardiac function is intentionally reduced by organ isolation in the Langendorff technique, it is unlikely that the acute removal of sex hormone signaling will completely revert structural and functional changes consequent of a lifetime of differences in endogenous sex-hormone signaling. Other sex-dependent differences without a clear underlying etiology include age-related changes. For instance, left ventricular (LV) systolic function, as measured by global longitudinal strain, is significantly higher in old versus young male mice but relatively preserved in females (52). Similarly, although both male and female mice have changes in aortic structure with age, only the males have concomitant changes in aortic function (52). Taken together, these studies suggest that care should be taken in considering the role of sex in studies using the Langendorff technique, particularly when used to analyze the effects of Ca^2+^ regulation, sex hormone signaling, and cardiac ultrastructural remodeling. Of note, there is a reason for growing optimism as a recent manuscript by Woitowich et al. (53) showed a significant increase in sex-specific reporting in general biology studies between 2009 and 2019.

#### Age.

In the manuscripts reviewed, 40.8% (*n* = 183) quantitatively reported animal age at the time of the experiment (Table 2). However, the majority of studies qualitatively reported using “adult” or “mature” animals. A recent survey among scientists found that “adult” was loosely defined as anywhere between 6 and 20 wk in laboratory rodents (54). These manuscripts were classified as “data not specified” for the animal age field because of the ambiguity associated with the qualitative descriptor. One factor likely contributing to the underreporting of animal age may be linked to age tracking by commercial vendors. For example, when our group requests the vendor to provide date of births for guinea pigs, the dates for all animals are identical in each shipment, which is possible, but unlikely when repeated weekly, and year after year.

The effect of age on cardiac structure and function has been examined extensively (55–60). From birth to adolescence, there are numerous and substantial changes to the heart. Structurally, cardiomyocytes increase in volume (hypertrophy), gaining their distinct rectangular shape, and proteins such as connexin43 and the voltage-gated sodium channel (Na_v_1.5) redistribute from a uniform sarcolemmal distribution at birth to a polarized distribution clustering at intercalated disks by the time of sexual maturity (55–57). Functionally, neonatal hearts demonstrate increased heart rate, faster atrioventricular conduction, shortened action potential duration, and decreased ventricular refractoriness relative to their adult counterparts in Langendorff-perfused Sprague–Dawley rats (61). With advancing age, there is an increase in fibroblast infiltration, reduction in vascular integrity, and loss of metabolic flexibility (62–64). These changes result in lower diastolic and mean arterial pressures, reduced isovolumetric relaxation time/diastolic time intervals, and elevated left ventricular end-diastolic and end-systolic volumes (52). Advanced age is a known risk factor for cardiac disease (59), and as the geriatric population of the world continues to grow, there is an increased need to refine our understanding of age on cardiac function (65–67).

While it is unlikely that age discrepancies of a few days to a week will substantially alter functional outcomes in adult animals, even nuanced differences in age could have a dramatic impact during early life (56, 57). For instance, between birth to 10 wk postnatal, there are rapid and significant changes in the orientation of transmembrane proteins on the cardiomyocyte, the innervation of the myocardium, and the metabolic biochemistry of the heart (55–57, 68, 69). In the rat, there is a 6.2-fold increase in LV weight and a 69% increase in septum secundum length and width within the first 11-days postnatal, and myocyte cell volume increases up to 25-fold in the first 2 months of life (70–72). In humans and rodents alike, the innervation of the heart is relatively immature at birth and continues developing during childhood and early adolescence (68, 73–75). Regarding energy metabolism, there is a switch from preferential carbohydrate dependence to fatty acid oxidation within the first 30-days postnatal in rodents; in humans, this switch occurs before birth (76–78). There are also significant increases in creatinine and phosphocreatine concentrations within the first month of life in dogs, rabbits, guinea pigs, mice, and rats (78, 79). Furthermore, geriatric aging is known to underlie substantial changes in cardiac structure and function. For instance, there is an increase in fibrotic infiltration, a decrease in vascular integrity, and dehiscence of the intercalated disk that occurs with geriatric aging (62, 63, 80). In the context of the Langendorff technique, Waldeyer et al. (81) demonstrated significant changes in action potential morphology and epicardial conduction velocity associated with aging in wild-type Langendorff perfused mouse hearts.

#### Weight.

Animal weight was quantified and reported in 25.4% (*n* = 114) of manuscripts reviewed. Table 2 shows reporting trends for the most frequently used animal models. Laboratory animal weight is a highly variable parameter, and one study noted that it is not uncommon for animals to arrive with weights 10% lower than noted by the vendor at the time of shipment (82). This weight difference may be explained by scale differences and/or weight loss during shipment. In particular with retired breeder guinea pigs, a geriatric model, animal weight can vary by upward of 5% daily depending on eating and hydration habits, defecation and urination differences, and whether fluids have been administered.

Changes in weight, particularly for prey species, are an indicator of emotional and physical stress (83). This becomes important when studying cardiac function, as both acute and chronic stress impact cardiovascular dynamics. For instance, the neurocardiac axis provides a framework for how acute emotional stress, as may occur with animal transportation, is linked with acute cardiovascular events. Sympathetic hyperactivation induces cytokine release and a proinflammatory state, leading to endothelial activation and potentially thrombosis in predisposed animals (84). Alternatively, chronic stress has been shown to impair baroreflex, chemoreflex, and heart rate variability, potentially hindering the study of coronary pressure and intrinsic heart rate (85).

Aside from obesity studies, there is no suggestion that small fluctuations in laboratory animal body weight, not associated with stress, will significantly alter experimental outcomes. However, body weight may give insight into organ weight, more specifically heart weight. This is of importance as higher cardiac/total body weight ratios have been linked with increasing likelihood of cardiac lesions and worse cardiovascular outcomes (86). This phenomenon was reviewed in a recent manuscript by Patel et al. (87), summarizing the effects of epicardial adipose tissue on cardiac function. As such, many studies continue to report organ weight (including heart weight) to body weight ratios, and therefore a general knowledge of animal weight ranges would be beneficial for cross-study comparisons (88, 89). In this regard, 27.3% (*n* = 121) of manuscripts specifically reported heart weights (31 manuscripts reported heart weight in grams only, whereas the other 90 reported heart weight-to-body weight or heart weight-to-tibia length ratios). It is also worth noting that “control” laboratory rodents are metabolically morbid compared with their undomesticated counterparts, presenting with a higher incidence of obesity, sedentary lifestyle, and insulin resistance (90).

In total, 19.6% (*n* = 88) of manuscripts reported both age and weight of the animal at the time of the experiment (Table 2). Often either age or weight is documented, but infrequently both, when characterizing laboratory animals. Although there are barriers to age tracking in laboratory animals, particularly those purchased from commercial vendors, a better understanding of specific age ranges would help inform the applicability of the research outcomes.

### Anesthetic Agent

Over the past several decades, numerous studies have shown that sedatives and anesthetics commonly used in animal research produce a wide range of off-target effects with significant impacts on cardiovascular function (91–96). Below, we briefly comment on some of the known cardiac effects of pentobarbital, ketamine, and isoflurane—the three most commonly reported anesthetics in the manuscripts reviewed (Table 3).

**Table 3.** Description of commonly administered anesthetics broken down by animal species distribution

	Chicken	Dog	Guinea Pig	Hamster	Mouse	Pig	Rabbit	Rat	Sheep	Trout	Totals	%
Ketamine	0	0	0	0	11	4	18	38	0	0	71	15.8
Pentobarbital	0	2	8	1	42	0	11	89	0	0	153	34.1
Isoflurane	0	1	5	0	18	0	3	22	0	0	49	10.9
Thiopental	0	0	0	0	0	0	1	17	0	0	18	4.0
Tribromoethanol	0	0	0	0	8	0	0	0	0	0	8	1.8
Chloral hydrate	0	0	0	0	2	0	0	11	0	0	13	2.9
Cervical dislocation/Decapitation	1	0	1	0	10	0	1	8	0	1	22	4.9
Other	0	0	0	0	3	1	0	16	0	0	20	4.4
Not specified	0	1	1	0	31	6	4	51	1	0	95	21.2
Totals	1	4	15	1	125	11	38	252	1	1	449	100.00

#### Pentobarbital.

Of the manuscripts reviewed, 34.0% (*n* = 153) used pentobarbital as their primary anesthetic agent. Pentobarbital is a short-acting barbiturate that agonizes GABA_A_ receptors, in turn, hyperpolarizing neurons within the central nervous system leading to dose-dependent sedation and depression of the sensory cortex (91). Several studies have explored the effects of pentobarbital on cardiac function in ex vivo heart preparations (92, 93). Segal et al. (92) demonstrated that in male Sabra rats, use of pentobarbital as the primary anesthetic, before cardioectomy, decreased the sensitivity of electrical and mechanical function to changes in extracellular calcium concentration (range = 0.5–2.5 mM) and thyroid hormone exposure (1 µM 3,5,3'-triiodothyronine for 0–40 min incubation). Jiang et al. (93) showed that pentobarbital (50 mg/kg) significantly decreased heart rate, left ventricular systolic pressure (LVSP), and the maximum rate of rise in left ventricular pressure (+*dP/dt*) in isolated Sprague–Dawley rat hearts when compared with nonanesthetized control rat hearts. The authors suggest that their results are due to a disruption of physiological calcium handling by pentobarbital. Taken together, these reports indicate that the use of pentobarbital may complicate comparisons of calcium handling and mechanical function between studies using other anesthetics.

#### Ketamine.

Ketamine was the primary anesthetic agent used in 15.8% (*n* = 71) of manuscripts in our analysis (Table 3). Ketamine is a noncompetitive *N*-methyl-D-aspartate (NMDA) receptor antagonist. NMDA inhibition in the central nervous system results in depressed sensation in the sensory association area of the cortex, the limbic system, and the thalamus (94, 95). Neuronal inhibition in these areas of the central nervous system results in an inability to receive and process sensory information, such as pain (94, 95). In addition, ketamine results in an analgesic effect via inhibition of nitric oxide (NO) synthase (96). An important off-target consequence of NO inhibition, relevant to the Langendorff technique, is that NO is involved in intrinsic vasoregulation (97). Similar to pentobarbital, Jiang et al. (93) reported that ketamine (100 mg/kg) significantly decreases heart rate, LVSP, and +*dP/dt* in isolated Sprague–Dawley rat hearts when compared with nonanesthetized control rat hearts. Furthermore, Sloan et al. (98) showed that ketamine protects against ischemia-reperfusion injury in a dose-dependent manner. Together, these reports suggest that the use of ketamine as an anesthetic agent may complicate broad interpretation of experiments in which intrinsic vasoregulation is important, or in studies investigating ischemia-reperfusion injury.

#### Isoflurane.

Of the manuscripts reviewed, 10.9% (*n* = 49) used isoflurane as their primary anesthetic agent (Table 3). Isoflurane, first discovered in 1965, is a methyl ethyl ether that is widely used for anesthetic purposes in clinical and preclinical studies alike (99). Unlike the aforementioned anesthetics, isoflurane is a volatile gas that is inhaled rather than injected, often in an induction chamber, or using forced ventilation with oxygen. Although the precise mechanism of action with isoflurane remains unknown, the compound produces a generalized, reversible depression of the central nervous system (100). Recent studies have demonstrated that isoflurane pharmacologically preconditions the mitochondria against ischemia-reperfusion injury by priming mitochondrial K_ATP_ channels via protein kinase-C signaling pathways, resulting in a blunted ischemia-reperfusion injury (101). Isoflurane has also been shown to attenuate reactive oxygen species produced by mitochondrial complex I and complex II substrates (102). Together, these reports suggest that isoflurane may uniquely affect mitochondrial energetics relative to injected anesthetics, particularly in the context of ischemia-reperfusion injury.

The changes in cardiac physiology accompanying the use of pentobarbital, ketamine, and isoflurane demonstrate that the choice of anesthetic could impact data interpretation and study conclusions. Of concern, 21.2% (*n* = 95) of manuscripts reviewed did not include details on anesthesia in their methods—creating a potential source for ambiguity in the interpretation and assimilation with related literature.

### Heparin

Of the manuscripts reviewed, 53.3% (*n* = 236) reported the injection of heparin before euthanasia; reported doses ranged from 1 U/kg to 5,000 U/kg. Furthermore, 2.0% (*n* = 9) of manuscripts specifically stated they did not use heparin before euthanasia, and the remaining 44.7% (*n* = 198) manuscripts did not specifically state whether heparin was or was not administered. Numerous studies have demonstrated the effect of heparin on cardiovascular function in laboratory animals. Specifically, heparin has been suggested to increase breakdown of vascular endothelial glycocalyx in rat models of ex vivo lung perfusion and to augment the vasopressor effect of protamine in conscious dogs (103, 104). In neither instance were the groups studying the direct effect of heparin on the heart, but their combined results suggest that heparin may alter vascular endothelial function, perhaps leading to differences in coronary artery function in Langendorff-perfused hearts. Though, more detailed studies are needed to positively determine the effect, if any, heparin administration has on ex vivo cardiac function.

### Cannulation Time

Of the manuscripts reviewed, only 2.5% (*n* = 11) quantified the time elapsed from excision to cannulation of the isolated heart, with times ranging from <30 s up to 4 min. Most manuscripts addressed cannulation time with a qualitative statement to the effect of, “the heart was quickly excised and mounted on a Langendorff apparatus.” To date, to our knowledge, no studies have directly examined the relationship between the duration of precannulation ischemia time (i.e., time from cardioectomy to cannulation and restoration of perfusion) and subsequent cardiac function.

An evaluation of the ischemia preconditioning literature may offer insights into how precannulation ischemia can influence cardiac function in hearts studied with the Langendorff technique. First described in 1986, ischemic preconditioning is an endogenous protective mechanism in which early periods of transient ischemia condition the heart to better withstand a subsequent ischemic insult and thereby provide protection against the threat of prolonged ischemia (105). The effect has been confirmed in all species studied, but the precise mechanism(s) by which preconditioning confers cardioprotection is an active area of research. What is known though is that the degree of cardioprotection is directly related to the duration of ischemic preconditioning (106).

In studies using the Langendorff technique, the time to cannulation is effectively an ischemic preconditioning period. Unintended consequences of precannulation ischemic conditioning are not limited to confounding results in ischemia or ischemia-reperfusion studies. For instance, one known consequence of preconditioning is increased activation of nitric oxide (NO) synthase and subsequent NO generation, which is known to have a vasodilatory effect within the heart (97, 107). The role of NO in vasospasm, as it relates to angina and ischemia, is also an area of significant research that might be impacted by precannulation ischemia (108). Furthermore, NO can modulate voltage-gated sodium channel conductance in cardiomyocytes by cyclic GMP and cyclic AMP pathways, highlighting a third direct role of NO on cardiac electrophysiology (109). As such, NO levels can impact the outcome of hemodynamic (110, 111) and electrophysiological (112, 113) studies performed using the Langendorff technique.

Recent guidelines by Bøtker et al. (14) recommend a cannulation time of <3 min for all hearts mounted with the Langendorff technique. Establishing an upper cannulation time limit is a complex task as extrapolating the necessary ischemic duration to induce preconditioning is difficult to ascertain from previous manuscripts given the wide range of reported times, with estimates spanning from 20 s to 15 min (114, 115). Given the functional effects of different anesthetics (see subsection “*Anesthetic Agent*”) and the sex-dependent differences in response to ischemia (116–118), it is foreseeable that establishing an upper limit to cannulation time will require multiple considerations and approaches. Increasing the accuracy of reporting cannulation times may therefore provide metadata that can inform future research in this area.

### Circadian Considerations

Although the time of surgery relative to the circadian cycle of animals was not noted in the manuscripts evaluated here, it may become a parameter of interest for a few reasons. First, the circadian cycles of activity are not consistent between species (119). For instance, humans are diurnal, guinea pigs are crepuscular, with activity mostly at dawn and dusk with rest periods in between and mice, rats, and hamsters for example are nocturnal (120). Performing experiments during the daytime, particularly if the animal facilities’ light/dark cycles are tied to the environmental day/night cycle, may yield results in these rodents that are more pertinent during human periods of inactivity (aka sleep), as has been well described in the oncology literature (121). Second, circadian mechanisms have been shown to govern cardiovascular function and arrhythmia susceptibility in mammals (122, 123). At the cellular level, outward potassium channels have been shown to display circadian variation, likely through their interaction with the KV channel-interacting protein 2 (KChIP2) (122). As interest in chronobiology increases, with the goal of optimizing therapies or identifying the circadian rhythms that increase the risk of death, knowing when an experiment was performed is an important consideration for improving rigor and reproducibility.

### Perfusate

#### Composition.

**Figure 1.**
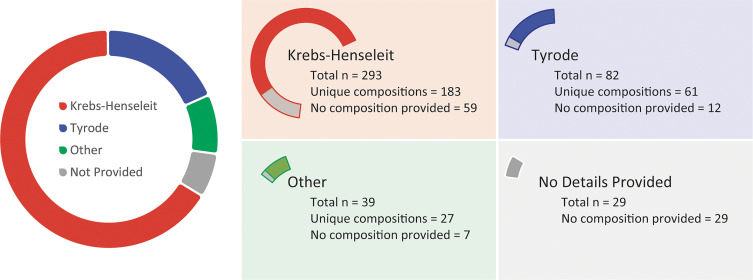
Breakdown of perfusates used by naming convention.

**Figure 2.**
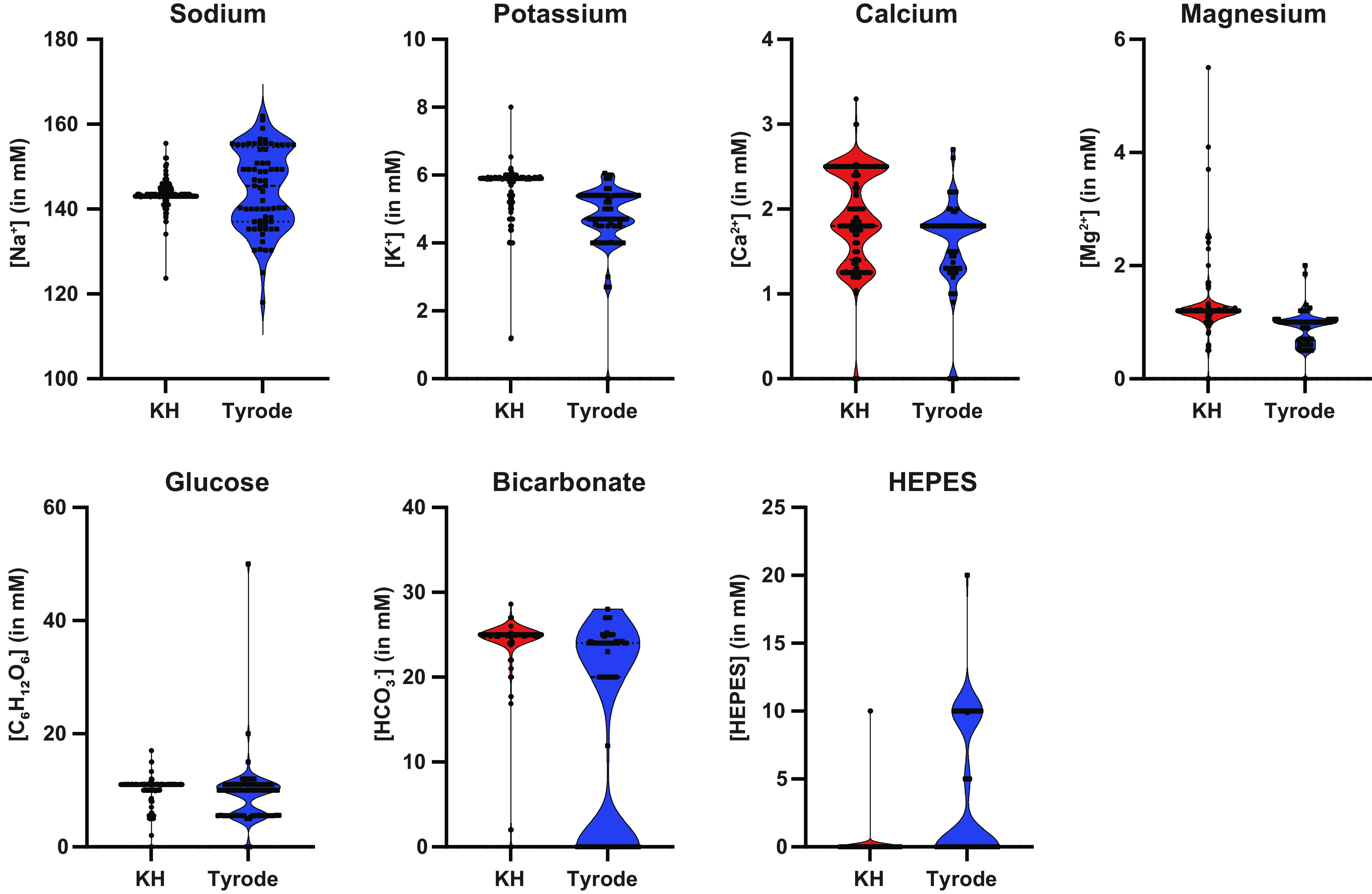
Violin plots of components commonly included in KH and Tyrode solutions. These components were selected for display based on their nearly ubiquitous appearance in perfusates reviewed. It is worth noting, that 59 manuscripts using “KH” and 12 manuscripts using “Tyrode” provided no details on perfusate composition and thus are not included in these violin plots. KH, Krebs–Henseleit.

In the manuscripts reviewed, 66.1% (*n* = 293) reportedly used a Krebs–Henseleit (KH) or “modified KH” solution, and 18.5% (*n* = 82) used a “Tyrode” or “modified Tyrode” solution (Fig. 1). The remaining manuscripts used a Krebs–Ringer solution (*n* = 10), blood supplemented perfusate solution or blood (*n* = 5), Ringer-Locke solution (*n* = 1), generically named “buffer/perfusate solution” (*n* = 23), or provided no details for their solution composition (*n* = 29). In this review, modified versions of KH or Tyrode are combined for analysis of KH solution or Tyrode solution composition, respectively. Of the 293 manuscripts using the KH nomenclature, 59 (20%) did not provide details of the solution composition. Of the remaining 234 KH solutions, there were 183 (75%) distinct perfusate compositions. Despite these composition variances, all are referenced by the same name. Similarly, of the 82 manuscripts using the Tyrode nomenclature, 12 (15%) did not describe the solution composition. In the remaining 70 manuscripts, there were 61 (87%) distinct perfusate compositions referenced by the same name. To demonstrate the variability existing within and between KH and Tyrode solutions, the distribution of sodium, potassium, calcium, magnesium, glucose, bicarbonate, and HEPES concentrations for all reported KH and Tyrode solutions are plotted in Fig. 2. These particular components were graphed because they are commonly included in all solutions. The graphs demonstrate substantial variation between KH and Tyrode solutions, but also within KH and Tyrode solutions.

Although the Langendorff technique has been adapted to answer a variety of experimental questions since its inception, the basic principles behind the technique have not changed in the past 125 years. However, the perfusion fluids paired with the Langendorff technique have evolved substantially. Despite the multitude of perfusate compositions existing in the current literature, naming conventions are such that nearly all perfusates are named a KH, Tyrode, or a modified solution. This is notable considering that neither the KH solution nor Tyrode solution were conceived for maintenance of ex vivo cardiac function.

For example, Maurice Vejux Tyrode, a pharmacologist at Harvard Medical School, published his research investigating the mode of action of purgative salts on the isolated rabbit intestine in 1910 (124). Tyrode’s solution was born out of necessity when he found that the perfusates previously described by Ringer, Locke, Hedon, and Adler all failed to viably sustain the perfused intestine (124). The KH solution, first published in 1932, was developed by Hans Krebs and his medical student Kurt Henseleit as a synthetic medium intended to approximate the composition of blood plasma to facilitate the study of urea formation in liver slices (125, 126). Since their inception, both solutions have been modified component by component to suit a myriad of experimental needs without a concurrent change in nomenclature to reflect these modifications (127).

An appreciation that altering perfusate ionic composition affects cardiac function dates back to the early 1880s when Ringer reported the essential nature of calcium in creating cardiac contractions (128–134). Since Julius Bernstein’s introduction of the “Membrane Theory of Electrical Potentials” in 1902, a substantial amount of work has been devoted to understanding how deviations in physiochemical gradients alter bioelectric events (135). During the 20th century, work by numerous biophysicists, physiologists, and physicians established our understanding of electrophysiology of excitable tissues, mechanical properties of contractile tissues, and metabolic function in living cells; in each case demonstrating the ability for changes in ionic gradients to influence cellular function (125, 126, 136–142). In recent years, studies investigating the effect of ionic control of cardiac function demonstrate that relatively modest changes in perfusate potassium, sodium, and calcium ionic concentrations have a profound effect on cardiac function under both physiological and pathophysiological conditions (143–151).

Although the traditional naming conventions lend themselves to preserving the names of scientists that have contributed seminal findings, the convention limits communication of the actual fluid compositions used in ex vivo isolated heart preparations. To eliminate ambiguity from methods reporting, the scientific community could shift to defining their solutions by components and concentrations as opposed to historical naming conventions alone.

#### pH.

In the manuscripts reviewed, 55.6% (*n* = 246) of studies specifically included information regarding the final pH of their solution, though <5% of manuscripts specified how the solution pH was equilibrated (e.g., titration of HCl, NaOH, or CO_2_). The latter is noteworthy considering that equilibration will change the total ion concentration of several solution components, potentially altering cardiac function (see *Composition*).

As early as 1880, the effect of pH on cardiac function was recognized when Walter H. Gaskell demonstrated that decreasing extracellular pH had a negative inotropic effect on isolated frog hearts (152). In the subsequent 139 years, many studies have determined the mechanisms by which acidosis, both intracellular and extracellular, modifies cardiac function (153–159). For instance, decreased pH has a significantly negative inotropic effect on excitation-contraction coupling pathways (160). Acidosis results in reduced transmembrane calcium current, calcium release from the sarcoplasmic reticulum, sensitivity of the contractile myofilaments to calcium, calcium uptake by SERCA2a, O_2_ consumption, and in turn, decreased metabolism and levels of high-energy transfer molecules such as phosphocreatine (155, 158). Acidosis is also known to decrease heart rate, increase the PR interval, and prolong the AV node refractory period (156, 161, 162). Conversely, alkalosis is associated with decreased T-wave amplitude and decreased pacing threshold (163). Increasing pH has also been identified as mediating interaction between the α_1_-adrenergic receptors and insulin receptor family in cardiomyocytes (164). In addition to the absolute value of pH, the choice of the buffer will also affect Langendorff perfused hearts. Specifically, exclusion of bicarbonate in HEPES-buffered solutions changes kinetics of the Na^+^-HCO_3_^−^ symporter, which has a direct role, via HCO_3_^−^ transport across the membrane, and an indirect role, via changing local sodium gradients and thus influencing the Na^+^-H^+^ transporter, in regulating intracellular pH in the heart (165–169). Furthermore, tuning pH is often achieved by adding NaOH; this additional sodium may affect Na^+^/K^+^-ATPase pumps, Na^+^ gradient, and other Na^+^-dependent ion channels within cardiomyocytes (170). Because of the multitude of transporters and ion channels that are dependent on not only the absolute hydrogen concentration but also the components that come together to achieve a certain pH, precisely documenting these during perfusion may allow a greater understanding of cardiomyocyte function and offer insights into heterogeneous outcomes.

#### Temperature.

In the literature reviewed, 75.2% (*n* = 333) of studies reported perfusing hearts at physiological temperature (37°C ± 2°C), 2.9% (*n* = 13) at below physiological temperature (ranging from 4°C to 35°C), and 21.9% (*n* = 97) did not specify the temperature at which the heart was maintained. Although the majority of studies were performed at, or near, physiological temperatures, the details surrounding temperature control were often absent. Of the manuscripts analyzed, 27.1% (*n* = 120) stated that excised hearts were rinsed in “ice cold” perfusate, one manuscript stated that the heart was rinsed in perfusate maintained at 37°C, and in the remaining manuscripts it was difficult to ascertain whether or not freshly excised hearts were rinsed in ice-cold perfusate, room temperature perfusate, or physiological temperature perfusate before cannulation, or no perfusate at all. It was also often unclear whether epicardial temperature was maintained consistent with coronary temperature by way of a heated humidity chamber or superfused in a heated bath or left free hanging at room temperature. Finally, 18 manuscripts specifically stated they recirculated perfusate, 20 manuscripts specifically stated they did not recirculate perfusate, and in the remaining 405 manuscripts it was unclear whether or not perfusates were perfused in a single pass system, where perfusate passes through the heart and is siphoned off as effluent waste, or a closed system where the effluent is collected and recirculated again through the heart. Recirculation is often used for studies involving expensive reagents, but care should be exercised to ensure that the technique does not substantively create conditions of physiologically relevant metabolite waste accumulation and pH imbalance. One way to address confounding variables introduced by recirculation could be the inclusion of open and recirculated vehicle control studies, if feasible.

At hypo- and hyperthermic temperatures, there are substantial changes in channel kinetics, ion flow, pH, and molecular processes (171–174). Both elastic and viscous components of cardiac muscle mechanics, the latter more so, increase with temperature (175) and may impact studies of hemodynamics with the Langendorff technique. Membrane potential, conduction velocity, and action potential magnitude are proportional to temperature, whereas action potential duration is inversely related to temperature (176, 177). One example of a temperature:variable relationship that is not bidirectional is from Vostarek et al., who observed a significant decrease in the amplitude of calcium transients with increasing temperatures from 37°C to 40°C but found them unchanged when decreasing temperatures from 37°C to 34°C (178). Of note, hyperthermic temperatures produce a significant increase in the rate of calcium release from the sarcoplasmic reticulum (179). While controlling endocardial, epicardial, and coronary temperature simultaneously and uniformly is a complex task, including temperature settings in methods reporting is recommended.

### The Citation Trail and Paywalls

While relying on citations to fully describe methods is an efficient way to keep the total word count below publication limits, it can lead to ambiguity, confusion, and/or inaccuracy. This is particularly the case when a text states “methods as previously reported” with modifications or deviations that are not clearly enumerated. Reliance on “methods as previously reported” is particularly troublesome when the citing and cited manuscripts include fundamental differences in approach, such as the utilization of different animal models. For instance, one manuscript using male Wistar rats states, “Ischemia/reperfusion was studied ex vivo according to the Langendorff model, with minor modifications (citation).” The paper cited referred to a manuscript in which Langendorff-heart perfusion was performed in a rabbit model. Generalizations such as these create ambiguity regarding anesthesia dosing, coronary flow rate or pressure, isovolumetric balloon size, etc., as it is unlikely that such parameters will remain constant when switching from a rabbit model to a rat model or vice versa.

A second limitation of relying on citations for methods reporting is the matter of access. With the recognition of the need for increased transparency and data sharing to improve reproducibility in science, open access journals are gaining in popularity. As we limited our review to open access manuscripts indexed in PubMed Central, it is worth noting that finding “methods as previously described” occasionally required looking outside of the open access literature. Citing previous methods in manuscripts that are not publicly available undermines the spirit of open access. To mitigate this, numerous journals are now making manuscripts available after some period of time, but this does not address the often acute need to understand a recently published manuscript. Hopefully, this review demonstrates that nuanced methodological details may be important in interpreting the results of a study and are a critical component of a rigorous and impactful scientific manuscript. One suggestion could be the creation of open access methodological reporting repositories that are maintained and updated by the authors/laboratory. Alternatively, just as abstracts are open access and indexed by PubMed, journals could consider making methods open access for studies that use frequently used methods.

### Strategies for Improving Transparency in Experimental Design

In the past decade, numerous manuscripts have provided guidance for improving rigor and reproducibility in multiple facets of preclinical cardiology (35, 180–183). While each of the aforementioned guidelines dives into a specific realm of preclinical cardiology, the narrative arc can grossly be distilled into the need for more transparent and standardized reporting practices; which should not be confounded with the need for strict standardization in experimental design that would have the potential to limit creativity and scientific exploration.

This manuscript adds to this narrative by detailing the wide range of methods associated with the Langendorff technique and advocating for more thorough methods reporting. While these assertions are based solely on the authors’ interpretation of the current state of the field, we suggest the inclusion of “methods tables” in manuscripts when using the Langendorff technique for primary data collection, with the hope that inclusion of “methods tables” can be adopted by journals and archived in an open access repository to inform future study design. Such a methods table could be established by a consensus report generated by a group of interested scientists, and include information on animal species, sex, age (when reliably attainable through vendors or bred in-house), and weight at a minimum. Furthermore, a “surgical considerations” table could be included that outlines the use and dosage of heparin (or lack thereof), the surgical anesthetic administered, any provided analgesic, the use of chilled/room temperature/or warm buffer for washing the excised heart, and total time to cannulation. Finally, a table containing information on perfusate composition and experimental agent(s) (drugs, peptides, etc.) could also be provided. One advantage of standardizing methods reporting in tables would be to reduce the creative lengths some go to avoid “self-plagiarism” in the methods. While well-intentioned, the continuous rewriting of methods can introduce copy errors. On the other hand, facilitating methods reporting with a table could also increase the likelihood that a critical change introduced into the experiment might not be noted if the writer is not forced to critically evaluate the methods section for each manuscript.

Tables could also establish a minimum information reporting practice for general Langendorff design, but there will always be a need for additional methods reporting for specific applications of the Langendorff technique such as optical mapping, left ventricular pressure recordings, ischemia-reperfusion protocols, etc., all of which may not be conducive to a tabular design. In these instances, we might suggest adding detailed experimental protocols to the supplemental materials that provide the reader with enough detail to not only understand the experiment but also replicate the experimental design precisely if so desired.

In all cases, if we share a joint goal of improving rigor and reproducibility, journals and reviewers alike should be cognizant of methods reporting during the manuscript review process and ensure inclusion of sufficient detail for each study. We appreciate that tables take up more physical space on the page relative to inline text and that the adoption of tables by journals may not be currently realistic. Instead, the aforementioned “open access methods” may be a first reasonable step to ensuring methods are freely available to all.

### Limitations of This Review

Our data provide only a recent snapshot of the Langendorff literature from 2017 to 2020, whereas a nondate constrained search of (Isolated[All Fields] AND (“heart”[MeSH Terms] OR “heart”[All Fields])) AND (Retrograde[All Fields] AND (“perfusion”[MeSH Terms] OR “perfusion”[All Fields]))) OR Langendorff[All Fields]) in PubMed and PubMed Central returns nearly 15,000 results spanning more than one century. As such, it must be noted that while our results can provide a glimpse into the recent past, they cannot describe the evolution of methods reporting as it relates to the Langendorff technique.

### Conclusions

Over one and a half centuries have passed since Ludwig and Cyon first published the technique of retrograde perfusion in ex vivo hearts. In the meantime, researchers have extensively used, and continue to use, the Langendorff technique for investigating the electrical, mechanical, and metabolic function of the heart. The Langendorff technique has been modified in a multitude of ways to probe questions across the spectrum of physiological and pathophysiological functions of the heart. As such, it would be inauspicious to review, in detail, all aspects of methods used for data collection in Langendorff prepared hearts. Instead, we described a subset of the experimental design that is conserved and most commonly documented in methods sections across Langendorff studies.

Numerous common areas of biological and experimental variation were detailed for consideration related to the Langendorff whole heart preparation, including animal models, anesthesia, cannulation time, perfusate composition, pH, and temperature. For each methodological variable, reporting practices were heterogeneous. Variations in these parameters may have an important impact on experimental outcomes in Langendorff-perfused hearts and serve as a potential explanation for why some studies are seemingly irreproducible. An alternative view, however, is that heterogeneous scientific outcomes are a desirable scientific product that can yield important insights into health and disease if methodological reporting is enhanced. In this regard, several attempts have been made at establishing guidelines for the design and reporting of cardiac electrophysiology studies (183, 184). Though, as the data above demonstrate, unreported variance remains a contemporary issue. As such, we must continue community discussions on methods reporting and work with journals to determine how to best approach methods reporting.

## GRANTS

This study was supported by the National Institutes of Health Grants F31-HL147438 (to D.R.K.), R01-HL141855, R01-HL138003, and R01-HL102298 (to S.P.).

## DISCLOSURES

No conflicts of interest, financial or otherwise, are declared by the authors.

## AUTHOR CONTRIBUTIONS

D.R.K., K.M.H., and S.P. conceived and designed research; D.R.K., K.M.H., G.S.H., and S.P. performed experiments; D.R.K., K.M.H., G.S.H., and S.P. analyzed data; D.R.K., K.M.H., G.S.H., and S.P. interpreted results of experiments; D.R.K., K.M.H., and S.P. prepared figures; D.R.K., K.M.H., and S.P. drafted manuscript; D.R.K., K.M.H., G.S.H., and S.P. edited and revised manuscript; D.R.K., K.M.H., G.S.H., and S.P. approved final version of manuscript.
